# Translation and Modification of a Mindful Eating Questionnaire for Children Assisted by Item Response Theory in Chinese Children and Adolescents

**DOI:** 10.3390/nu14142854

**Published:** 2022-07-12

**Authors:** Dan Wang, Yuzheng Hu, Hui Zhou, Zhihong Ye, Junfen Fu

**Affiliations:** 1 Nursing Department, Children’s Hospital, Zhejiang University School of Medicine, National Clinical Research Center for Child Health, Hangzhou 310052, China; 12018380@zju.edu.cn; 2 Department of Psychology and Behavioral Sciences, Zhejiang University, Hangzhou 310063, China; huyuzheng@zju.edu.cn (Y.H.); huizhouzju@zju.edu.cn (H.Z.); 3 Nursing Department, SirRunrun Shaw Hospital, Zhejiang University, Hangzhou 310016, China; 4 Endocrinology Department, Children’s Hospital, Zhejiang University School of Medicine, National Clinical Research Center for Child Health, Hangzhou 310052, China

**Keywords:** mindful eating, children, adolescents, questionnaire, item response theory

## Abstract

Mindful eating has gained attention in studies on healthy eating. However, measurement of it is scarce, particularly in pediatrics. This study aimed to translate and modify the 12-item Mindful Eating Questionnaire for Children (MEQ-C) using techniques based on both classical test theory (ICC) and item response theory (IRT) in Chinese children and adolescents. Of the 426 participants enrolled and randomly grouped, the test (*n* = 223) and validation (*n* = 203) subsamples were well-matched in age, gender, body mass index z score (BMIz), and waist to height ratio (WHtR) (*p* > 0.556). Three items were eliminated due to deviating from the mindful eating concept (content validity index < 0.71) and presenting as an independent dimension in parallel analysis, or yielding a poor distribution (−4.331 < b < −0.111). The final 5-item Mindless Eating and 4-item Awareness subscales were identified with sound Cronbach’s α of 0.802 and 0.779, respectively. The remaining items functioned well (a > 1, −3 < b < 3), and the Mindless Eating subscale was accurate for the low-to-medium range (−2 to 0) of the mindful eating measure. The Awareness one was reliable for the relatively high range (0 to 2). Participants’ mindful eating characteristics should be taken into consideration in practice.

## 1. Introduction

Mindful eating refers to eating with awareness and intentionally, focusing on physical and emotional sensations other than judgment. Due to less resistance to natural eating desires compared to dieting [1], mindful eating has been recognized as an effective intervention that could help obese people lose weight safely. In fact, a recent study has shown that mindful eating acts as effectively as dieting [2] and may produce a more persistent effect [3].

Accordingly, mindful-eating-related measurements have also been developed and evolved to assess mindful eating traits. One of the most popular measurements used is the Mindful Eating Questionnaire (MEQ) developed by Framson et al. [4] to measure the mindful eating style in adults. This includes five dimensions: disinhibition, awareness, external cues, emotional response, and distraction [4]. A higher total MEQ score indicates a higher level of mindful eating traits, which is found to be negatively associated with weight status in adults [4]. Although other measurements, such as the Expanded and Four-Facets Mindful Eating Scales or inventory are available for adults [5,6,7], the MEQ has been widely used as a primary mindful eating measurement in practice [7].

The promising effect of mindful eating on weight loss has drawn increasing attention for its benefits to children and adolescents. However, only limited mindful eating measurements are available for children and adolescents. Considering the high prevalence of overweight and obesity in children and adolescents globally and the unknown safety of the interventions based on diet [8], a number of studies have attempted to adapt the mindful eating interventions to pediatrics [9,10,11]. The results demonstrate the feasibility of teaching mindful eating to children and adolescents and that such a strategy does induce negative eating behaviors [10,12]. The measurements and interventions based on mindful eating have been of significance in verifying the effectiveness of mindful eating and cross-study comparisons. However, due to the lack of reliable measurements to characterize mindful eating behaviors, the outcomes of many mindfulness-based interventions have not been evaluated accordingly [3].

To facilitate mindful eating research and practice in pediatrics, Hart and colleagues [13] developed the Mindful Eating Questionnaire for Children (MEQ-C). The MEQ-C is a 12-item questionnaire adapted from the MEQ developed by Framson [13]. In contrast to the MEQ, the MEQ-C only has two subscales, Mindless Eating and Awareness [13]. 

However, the preliminary MEQ-C that was developed and tested based on classical test theory (CTT) may not provide sufficient information about the item/test difficulty and discrimination functions, or whether the test difficulty matched the ability levels of participants [14]. Moreover, the CTT-based results are also influenced by the different sample characteristics and do not effectively connect the individual characters with the latent trait [15]. In contrast to the CTT, which estimated the parameters based on the true scores from particular examinee samples in parallel test forms, the item response theory (IRT) assesses the item functions, such as the item difficulty and discrimination based on the response probability [14]. The IRT techniques could build an association between a latent variable and an individual trait, which could increase the test score validity by matching the test difficulty to the latent ability levels of the examinees [16]. This sample-free estimation character based on response probability could provide more accurate information when developing and modifying measurements 

In addition, further exploration of the association between mindful eating and weight status could verify the predictive validity of the measurement and be important for understanding the relationship between mindful eating and weight. This could provide more evidence for weight management programs on mindful eating. 

To narrow the gap of the problems discussed above and provide more accurate information for MEQ-C application, we translated MEQ-C into Chinese and modified it with psychometric property indicators based on both CTT and IRT. Consequently, we further explored the association between mindful eating and weight status characterized by BMIz and WHtR in children and adolescents.

## 2. Materials and Methods

### 2.1. Translation Process and Pilot Test of Preliminary MEQ-C-C

This study was part of a battery of eating-related measurement translation works conducted from November 2020 to June 2021. The Mindful Eating Questionnaire (MEQ) was translated into Chinese along with the dimensional Yale Food Addiction Scale for Children 2.0 (dYFAS-C 2.0) [17] and the Kids-Palatable Eating Motives Scale (K-PEMS-C) [18], etc. 

The questionnaire was translated via Beaton’s method [19]. The MEQ-C was translated into Chinese by two researchers and back-translated by another two researchers. All of the researchers conducted the translation works independently. After several rounds of translations and back-translations, a preliminary Chinese version of the Mindful Eating Questionnaire for Children (MEQ-C-C) was formed. Subsequently, this was sent to seven multi-disciplinary experts concerned with mindfulness or eating behaviors for content validity appraisal. In the item content validity analysis, item 9 and item 10 were not consistently endorsed (Item 9—When I am sad I eat to feel better; Item 10—When I am feeling nervous or worried I want a snack). The experts recognized that these two items refer to emotional eating, similarly to the items in the coping subscale of (K-PEMS-C). Although mindful eating also involves emotional issues, it has more to do with the emotional perceptions derived from the eating process, rather than the desire to eat due to emotional fluctuation. Thus, the inclusion of these two items was reconsidered through further psychometric tests. A total of 12 items were distributed to participants. The results of the content validity index (CVI) of the items were good enough, except for items 9 and 10 [20]; see Table A1.

A sample of 32 participants completed the 12-item version of MEQ-C-C and provided their feedback about the understanding of wordings. The participants highlighted that certain items required further explanation, such as the meanings of “I like the way my food looks on my plates,” and “I taste every bite of food,” in addition to “Sometimes I eat food just because it’s there.” Further, the participants were also asked to provide their suggestions for better expressions after the researchers explained the items they found obscure. In addition, the participants mentioned that the response category was rough. There were four categories: “Never/Rarely,” “Sometimes,” “Often,” and “Always/Usually.” There is an obvious difference between “Never” (0 times) and “Rarely” (happened occasionally), and the frequency of “Usually” was similar to that of “Often.” In addition, it might be improper to make usually and always (happens every time) a category. Subsequently, the MEQ-C-C was modified according to this feedback, and a formal version of MEQ-C-C was created with items rated by a 5-point Likert scale (never/rarely/sometimes/often/always).

### 2.2. Formal Validation 

#### 2.2.1. Participants

The participants aged from 8 to 18 years and had regular physical checks in the outpatient growth and development department of a children’s hospital. To be enrolled, the participants were required to be healthy, without any diagnosis, and come to the hospital to have their regular development checkups, such as height monitoring. Participants with disease diagnoses influencing eating behavior (such as hyperthyroidism or diabetes) and participants with mental disorders were excluded.

#### 2.2.2. Measures 

##### MEQ-C-C

The MEQ-C-C was translated from the MEQ-C developed by Hart [13]. A 5-point scoring replaced the 4-point approach in the original MEQ-C according to the participants’ feedback, and for better response accuracy [21]. Items 1, 2, 3, 8, 11, and 12 were reverse coded from Never (5) to Always (1), and items 4, 5, 6, and 7 were coded from Never (1) to Always (5). The average total scores were calculated for analysis, and higher scores indicated greater mindfulness in both subscales.

##### BMIZ and WHtR

The height, weight, and waist were measured by the researcher to calculate the BMIZ and WHtR of participants. The BMIZ score was calculated by the WHO AnthroPlus software. According to the WHO’s anthropometric calculator, BMIZ > +1 SD was defined as overweight and BMIZ > +2 SD as obesity [22]. The WHtR is an indicator of abdominal obesity. Boys with WHtR ≥ 0.48 and girls with WHtR ≥ 0.46 were deemed as having abdominal obesity [23].

#### 2.2.3. Procedures

The researcher measured participants’ height and weight. The participants completed the consent information online, followed by an online survey with a series of measurements. 

All participants were informed about the detailed information of the study. This project was approved by the Ethics Board Committee of Children’s Hospital of School of Medicine, Zhejiang University (2020-IRB-179).

### 2.3. Data Analysis

The survey sample was randomly split into two subsamples for the exploratory factor analysis (EFA) and the confirmatory factor analysis (CFA), respectively. The characteristics of participants were summarized using proportion, mean (standard deviation, SD), or median (inter-quartile range, IQR), and compared by Student’s *t*-test or non-parameter test when appropriate. The initial structure of the scale was explored by EFA in one sample and then validated by CFA in another sample. The reliability was indicated by internal consistency using Cronbach’s α and McDonald’s omega, and test-retest reliability using intra-class correlation coefficient (ICC). Subsequently, the graded response model (GRM) derived from item response theory (IRT) was used to test the properties of the remaining items of each independent subscale to explore the difficulty, discrimination, and test information. Pearson correlation and regression analysis were used to explore the association between MEQ-C-C and weight status. The statistical analyses were conducted by SPSS 23.0 and mirt in R. *p* < 0.05 was deemed significantly different.

## 3. Results

### 3.1. Demographic Information

A total of 426 participants aged from 8.03 to 17.35 (Mean = 10.53, SD = 1.68) completed the survey. Of the participants, 277 girls and 149 boys were included. The sample was split into two subsamples randomly using the SPSS procedure. No significant statistical differences were found between the two subsamples; see Table 1. Sample 1 was used to test the preliminary reliability and initial construct of the MEQ-C-C, and Sample 2 was used to validate the results from Sample 1 and to explore the association between MEQ-C-C and weight status. The participant characteristics of the two subsamples are listed in Table 1.

### 3.2. Preliminary Testing and Modification of MEQ-C-C with Sample 1

**Figure 1:**
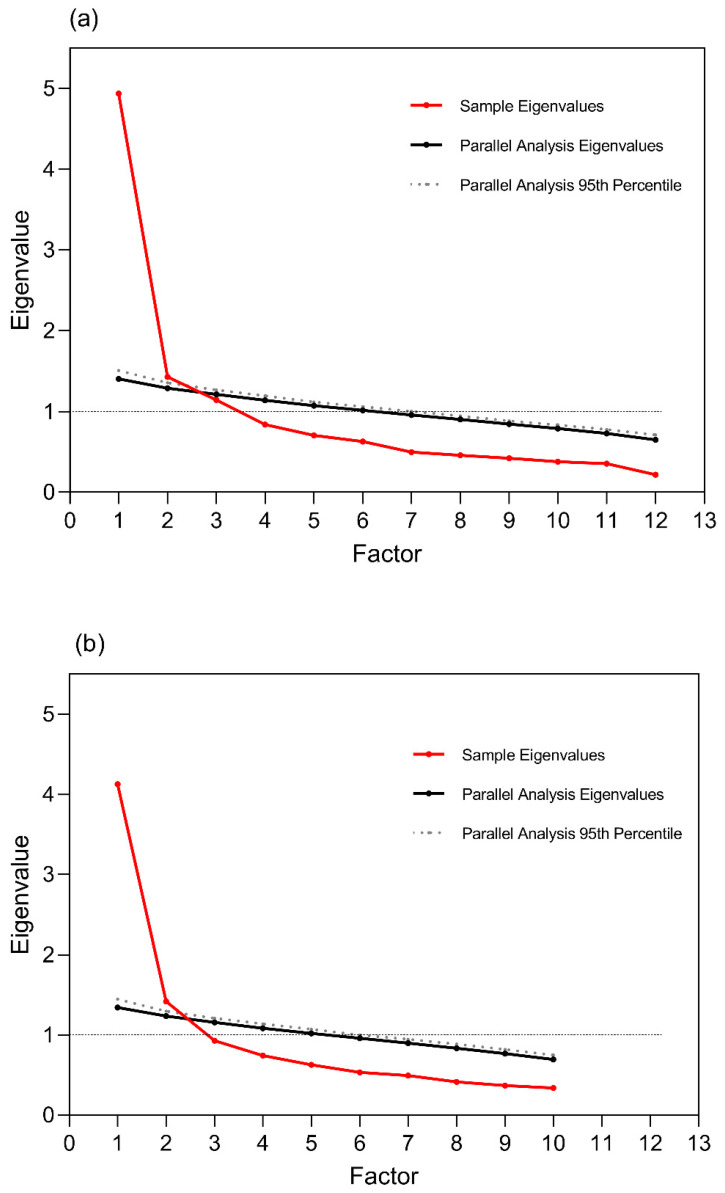
Parallel analysis of the MEQ-C-C. (**a**) Parallel analysis with 12 items; (**b**) parallel analysis without items 9 and 10.

As the original scale was a multi-dimensional measurement, the EFA was conducted firstly in Sample 1 to confirm the potential structure of the measurement. The parallel analysis was initially conducted using the minimum residual extraction method combined with oblique rotation to decide how many factors it was best to retain. The results indicated that three factors were strong, including Factor 1 (ME1, ME2, ME3, ME8, ME11, ME12), Factor 2 (ME4, ME5, ME6, ME7), and Factor 3 (ME9, ME10); see Table 2. Factor 1 and Factor 2 were consistent with the Mindless Eating subscale and the Awareness subscale of the original MEQ-C. Items 9 and 10, with relatively poor CVI in the previous part, were identified as an independent factor, which implied different components of measures compared to the other two factors. Moreover, according to Izquierdo’s study [24], poor factors defined by one or two items should indicate reconsidering the number of factors extracted. Further, the scree plot showed two-factor analysis was best suited, since only two factors were above the simulation line (Figure 1a). Thus, items 9 and 10 were finally deleted due to the conceptual inconsistency and the results of parallel analysis. The repeated parallel analysis with 10 items also supported the two-factor construct as well (Figure 1b). However, item 8 might need to be further inspected because it presented as an independent factor (Table 2). 

**Figure A1:**
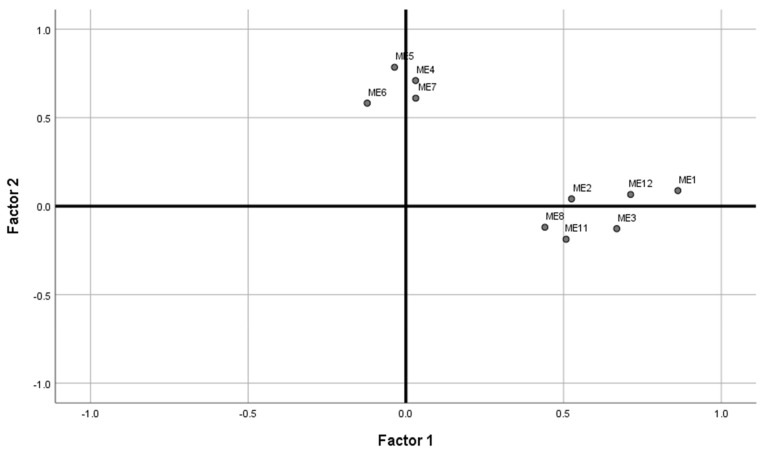
Component plot in rotated space.

To better explore the structure and compare the results with the original MEQ-C, the EFA of the 10-item using maximum likelihood estimator (ML) method with oblique ration as the original MEQ-C did was conducted. The Kaiser-Meyer-Olkin (KMO) was 0.85 (*p* < 0.001). Two factors were identified, which is consistent with the two subscales of the original MEQ-C. Factor 1 (ME1, ME2, ME3, ME8, ME11, ME12) and Factor 2 (ME4, ME5, ME6, ME7) were designated as Mindless Eating and Awareness according to the original MEQ-C (Figure A1). The loadings on factors of each item are presented in Table A2. The two factors could explain 45.2% of the variance. Interestingly, item 8 belonged to Mindless Eating subscale with a relatively low factor loading (λ = 0.441) compared with other items in Mindless Eating. Therefore, further inspection was conducted to explore the inconsistent results of item 8 in the parallel analysis (as a single factor). 

The internal reliability of the Mindless Eating and Awareness subscales were 0.802 (Cronbach α) and 0.811 (McDonald’s ω) and 0.779 (Cronbach α) and 0.782 (McDonald’s ω), respectively. See Table A3.

The ICC of Mindless Eating and Awareness were 0.392 and 0.379, respectively.

In the preliminary CFA with Sample 1, the model fit was acceptable in two subscales. However, in the Mindless Eating subscale, the Modification Index (MI) resulting from CFA indicated a potentially better model fit by removing item 8 (MI = 25.87) or item 11 (MI = 25.65). The two items were checked for their content. Item 8 was, “Sometimes I eat food just because it’s there”; item 11 was, “I have trouble not eating candy, chips, or cookies if I can have them.” Item 8 referred to eating without particular reason, and item 11 referred to difficulty constraining oneself from eating when facing delicious food. Item 8 seemed prone to illustrate eating motives (eating when bored).

### 3.3. Further Modification to MEQ-C-C Assisted by IRT

**Figure 2:**
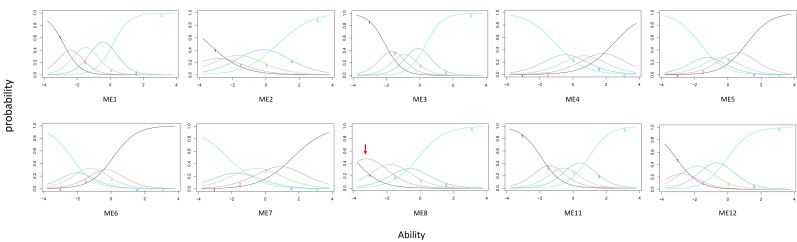
The response characteristic curves of items.

**Figure 3:**
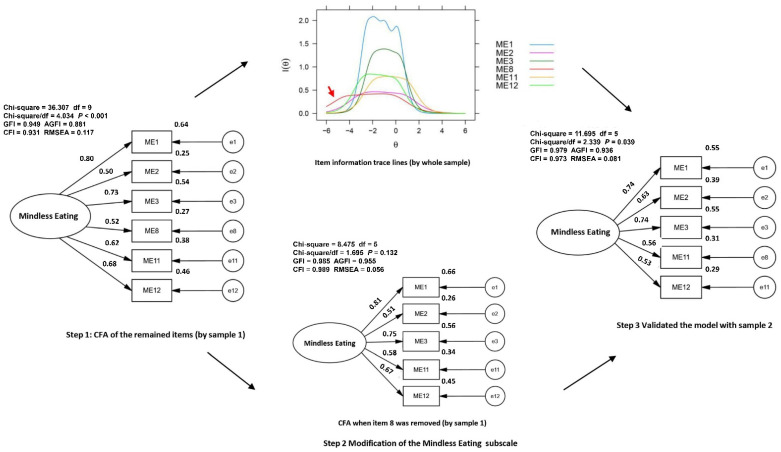
The modification and validation of the Mindless Eating Subscale assisted by item information trace lines.

To further test the items of the Mindless Eating subscale, particularly items 8 and 11, item characteristic analysis was conducted using GRM. The results indicated that the difficulty parameter of item 8 was out of the range of -3 to 3 according to the parameters of item function (Table 3) and did not yield an ideal response characteristic curve distribution [14] (one middle curve was out of normal distribution, Figure 2). In addition, the item information trace line (Figure 3), indicated that item 8 might require removal for better measurement. Consequently, item 8 was removed tentatively, and the results showed that Cronbach’s α of the 5-item Mindless Eating subscale was 0.787 and the McDonald Omega was 0.801. The model fit was excellent following the elimination of item 8 ($\chi^{2}$/*df* = 1.695, GFI = 0.985, AGFI = 0.955, CFI = 0.989, RMSEA = 0.056); see Figure 3. The remaining 5 items of the Mindless Eating subscale yield good functioning of targeted trait measure (a = 1.318–2.782; b = −2.939–1.009, Table A4). Therefore, item 8 was finally removed due to poor item function.

The CFA with Sample 1 showed the 4-item Awareness subscale had an acceptable model fit with less modification potential according to MI and previous test results: $\chi^{2}$/*df* = 5.522, GFI = 0.978, AGFI = 0.890, CFI = 0.963, and SRMR = 0.0362.

**Figure A2:**
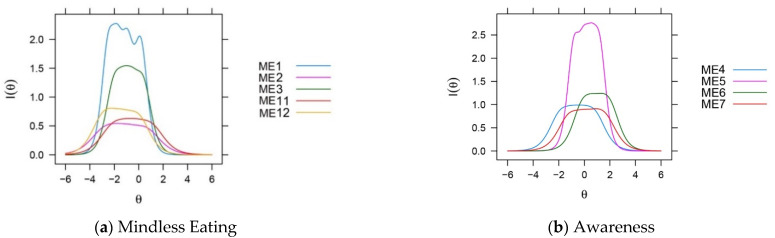
Function information trace line of Mindless Eating and Awareness subscales. (**a**) Item function trace of Mindless Eating subscale. (**b**) Item function trace of Awareness subscale.

Although the Awareness subscale did not yield an ideal model fit as the Mindless Eating subscale did, the results of the IRT analysis of the items in Awareness indicated that the four items functioned effectively (See Table 3). The function information trace line of Awareness is presented in Figure A2. These results show the items themselves in Awareness were good enough to test the awareness trait in eating. The dissatisfactory outcome in the CFA model fit may have been due to the unmatched trait level with the Awareness subscale in this sample.

**Figure 4:**
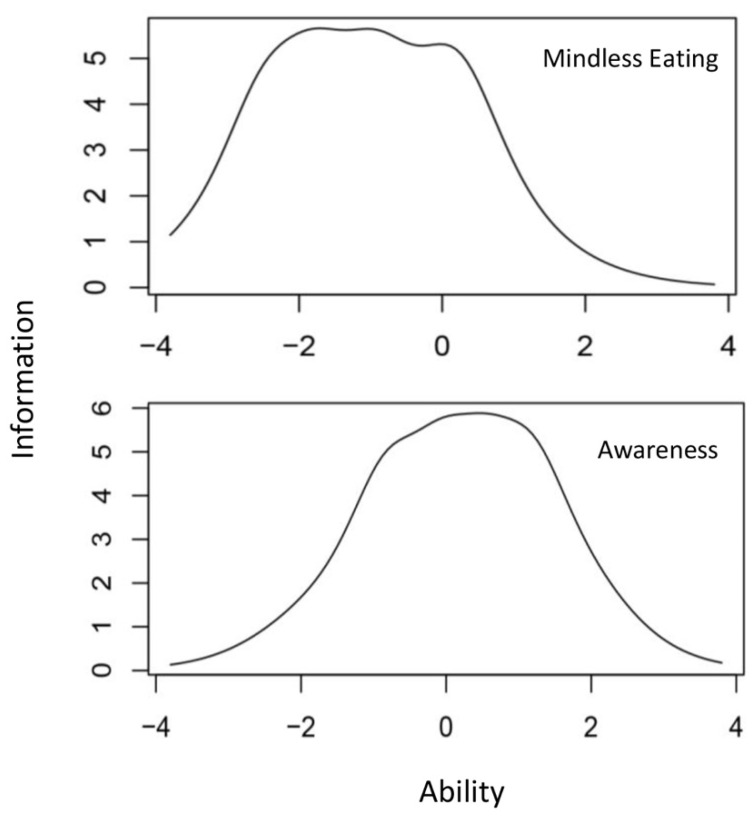
Test information trace lines of two subscales.

The results highlighted the remaining items of the two subscales yield sound property performance. The difficulty parameters all ranged between −3 and 3, indicating reasonable latent trait distribution [25]. The discrimination parameters being all above 1 showed these items could differentiate the different levels of latent traits [25] (Table 3). The information curve of measure suggested that the Mindless Eating subscale was best suited to the low-medium mindful eating profile population, and the Awareness subscale was more reliable when measuring individuals within the medium-high profile population (Figure 4).

### 3.4. Validating the Test Results in Sample 2

The CFA of 5-item Mindless Eating subscale was tested previously in the modification stage using Sample 2. The model fit was good (Figure 3), and its reliability was sound (α = 0.771, ω = 0.780). The model fit of the Awareness subscale was as follows: $\chi^{2}$/*df* = 6.685, GFI = 0.971, AGFI = 0.856, CFI = 0.952, and SRMR = 0.0414 (α = 0.790, ω = 0.793).

**Figure 5:**
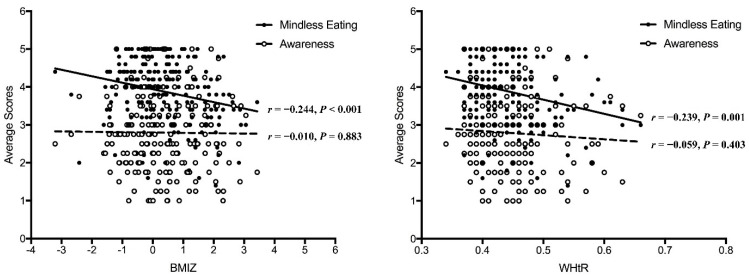
The associations between subscales and weight status. A case was excluded in the WHtR figure due to being an outlier (WHtR = 0.11, Mindless eating = 3.6, Awareness = 3).

The associations between the two subscales and weight status are presented in Figure 5. The regression analysis showed the Mindless Eating subscale was associated with BMIZ (B = −0.244, t = 3.570, *p* < 0.001) and WHtR (B = −0.239, t = 3.490, *p* = 0.001). However, the Awareness subscale was not significantly associated with any weight status index (t = 0.147, *p* = 0.883; t = 0.838, *p* = 0.403).

## 4. Discussion

This study translated the MEQ-C developed by Hart and validated it in Chinese children and adolescents. It was further modified by psychometric property indexes based on both CTT and IRT. The reliability of the two subscales of Mindless Eating and Awareness was sound. The items’ functioning in terms of discrimination and difficulty was good enough to measure the latent traits. The results highlighted that the Mindless Eating subscale was more reliable for individuals with low-to-medium mindful eating trait levels, whereas the Awareness subscale revealed more accurate test information for individuals with medium-to-high levels of mindful eating. The negative correlations between BMIz and WHtR, and the Mindless Eating subscale, support the potential of mindful eating interventions in children and adolescents. 

The two-subscale structure was aligned with the original version of the MEQ. However, in this study, items 9 and 10 were deleted after content validity discussion. This differed from Hart’s study, where the two items were distributed in the Mindless Eating subscale [13]. However, eating to cope with emotional episodes overlapped with the emotional eating motive measured in other tools, such as the K-PMES [18]. In another study conducted by Diana, the authors pointed out the items of “emotional response” in the original MEQ and emphasized the reaction to emotion in the eating situation, rather than awareness of the emotional trigger [7]. Here, the latter was presentative of the mindfulness in eating, but the former was not. 

Moreover, the internal consistency reliability of MEQ-C-C was significantly improved (α = 0.802) after the deletion of items 5, 6, 4, and 7. This showed the inconsistency of the original items. Reliability is the foundation of validity achievement, and it is important to have homogeneous items in the measurement [15]. The items deleted from the preliminary analysis happened to be fine in the other subscale, and sound internal reliability was found for these items. The results supported two distinct subscales and were consistent with Hart’s study [13]. 

The 5-item Mindless Eating subscale performed well, and mindful eating indicated by the Mindless Eating subscale was also negatively correlated with weight status, which revealed good predictive validity. These results provide further evidence for the effect of mindful eating on weight. This could be evidence for the construction of mindful-based eating interventions. 

However, the Awareness subscale was not as ideal as Mindless Eating. Although sound item function and internal reliability were acquired, the Awareness subscale did not indicate a good model fit within our sample. The results are similar to those in the original study of MEQ-C [13]. In addition, no significant association was found between the Awareness subscale and weight status, which did not yield a predictive validity. This may be because the expression salience of the Awareness subscale was not as obvious as that of the Mindless Eating subscale for children and adolescents. The items in Awareness consisted of positive statements to express mindful eating. However, the relatively new concepts of mindful eating may not be truly comprehended by the younger population. In addition, the test information in IRT analysis revealed that the Awareness subscale may perform well with individuals characterized by relatively high levels of mindful eating traits. Therefore, the children and adolescents might not be the ideal subjects for the Awareness subscale. This could explain why the sound reliability and content validity could result in an unexpected disassociation between Awareness and weight status. 

Although evidence from this study favored the independent use of the Mindless Eating subscale in children and adolescents, the results from IRT suggest that chosen measurements in different samples and situations should be taken into consideration. The relatively low level of mindful eating traits in our sample may partially account for the excellent performance of the Mindless Eating subscale. This is because, unlike the Awareness subscale, this measure could reveal most information within such a low-medium range sample. Therefore, further evidence from a different sample, especially of childrenwith different levels of mindfulness eating traits, is required for a better understanding of mindful eating measurement in children and adolescents. 

### Limitations

This study has several limitations. Firstly, in the IRT analysis, the subscales were analyzed separately rather than as a whole questionnaire of MEQ-C. Further study could be conducted based on the multi-dimension IRT approach to acquire more information about the MEQ-C-C. However, given the distinct differences between the two subscales, the GRM for each subscale may be suitable. Secondly, the sample from one single center may share similar latent mindful eating traits, which does not capture characteristics of different mindful eating levels. However, with the IRT analysis, the results provided us with the item and test functioning, which indicated more diverse samples for the MEQ-C-C validation. Thirdly, the generalization of the MEQ-C usage in different cultures was not validated in this study. This may not conclude comparable results across studies. The researchers were encouraged to work together for the invariance test of the MEQ-C. 

## 5. Conclusions

The MEQ-C consisted of two subscales: Mindless Eating and Awareness. The two subscales with good reliability could be used in populations with different levels of latent mindful eating traits. The Mindless Eating subscale revealed mindful eating in an opposite-stated way and was more readable and reliable among children and adolescents. In addition, the Mindless Eating subscale indicated an inverse correlation with weight status. The Awareness subscale could be suitable for individuals with relatively high levels of latent mindful eating traits. Further replication studies are required in populations with different characters.

## Figures and Tables

**Table 1:** The invariance comparison of the two subsamples.

Item	Sample 1 (*n* = 223)	Sample 2 (*n* = 203)	χ2/t/Z	*p*
Age	10.58 ± 1.67	10.48 ± 1.70	0.579	0.563
Gender			0.166	
Girl	80	69		0.684
Boy	143	134		
BMIZ	0.26 (−0.39, 1.12)	0.00 (0.00, 1.00)	−0.190	0.849
WHtR	0.45 ± 0.06	0.44 ± 0.06	0.589	0.556

Note: BMIZ: body mass index z score; WHtR: waist to height ratio.

**Table 2:** The factor loading resulted from parallel analysis.

Item	12 Items-Factor				10 Items-Factor			
	1	2	3	Uniqueness	1	2	3	Uniqueness
ME1	0.825			0.321	0.850			0.309
ME2	0.601			0.698	0.526			0.744
ME3	0.646			0.442	0.705			0.403
ME4		0.723		0.505		0.700		0.527
ME5		0.763		0.370		0.780		0.363
ME6		0.535		0.563		0.599		0.566
ME7		0.601		0.618		0.636		0.625
ME8	0.316			0.709			0.995	0.005
ME9			0.764	0.314		/		/
ME10			0.872	0.222		/		/
ME11	0.382			0.585	0.340			0.573
ME12	0.576			0.497	0.632			0.556

Note: MEQ-C-C = Chinese Version of Mindful Eating Questionnaire for Children.

**Table 3:** Estimation of item parameters of discrimination and difficulty.

Item	Factor Loading	a	b1	b2	b3	b4
Mindless Eating						
1	0.8	2.650	−2.523	−1.796	−0.931	0.161
2	0.5	1.217	−2.970	−2.041	−0.892	0.644
3	0.73	2.118	−2.063	−1.265	−0.561	0.362
8	0.52	1.164	−4.331	−2.579	−1.211	−0.111
11	0.62	1.595	−1.944	−0.984	−0.142	0.947
12	0.68	1.648	−2.899	−2.252	−1.235	−0.042
Awareness						
4	0.69	1.804	−1.876	−0.946	−0.106	0.782
5	0.81	3.051	−0.816	−0.050	0.556	1.170
6	0.65	1.996	−0.152	0.573	1.355	1.964
7	0.59	1.720	−1.204	−0.150	0.843	1.589

Note: a: discrimination parameter; b: difficulty threshold parameter.

## Data Availability

The data of this study are available upon reasonable request.

## Appendix A

**Table A1:** Content validity indexes of the MEQ-C-C.

Item	A	B	Experts C	D	E	F	G	Item-CVI
Item 1	4	3	4	4	4	4	4	1
Item 2	3	3	4	4	3	4	4	1
Item 3	3	4	4	4	3	4	4	1
Item 4	3	4	4	4	2	4	4	0.86
Item 5	3	4	4	4	2	4	4	0.86
Item 6	3	3	4	4	2	4	4	1
Item 7	3	3	4	4	2	3	3	0.86
Item 8	3	4	4	4	2	4	4	0.86
Item 9	2	3	3	3	2	3	4	0.71
Item 10	3	3	4	4	2	2	3	0.71
Item 11	3	4	4	4	3	4	4	1
Item 12	4	4	4	4	2	4	4	0.86
Proportion relevant	0.92	1.00	1.00	1.00	0.33	0.92	1.00	0.88

Note: MEQ-C-C = Chinese Version of Mindful Eating Questionnaire for Children; CVI = content validity index.

**Table A2:** The factor loading of each item after rotation.

Item	Component	
	1	2
ME1	0.862	
ME12	0.712	
ME3	0.669	
ME2	0.525	
ME11	0.508	
ME8	0.441	
ME5		0.785
ME4		0.710
ME7		0.610
ME6		0.582

Note: Only loading > 0.3 presented.

**Table A3:** Internal consistency reliability test.

Item	The Item-Total Scale Correlation Coefficient	Cronbach’s α If an Item Dropped	Cronbach’s α of Subscale
Mindless Eating			0.802
ME1	0.696	0.745	
ME2	0.415	0.804	
ME3	0.637	0.752	
ME8	0.484	0.787	
ME11	0.570	0.770	
ME12	0.586	0.766	
Awareness			0.779
ME4	0.589	0.723	
ME5	0.661	0.683	
ME6	0.554	0.741	
ME7	0.535	0.751	

**Table A4:** Estimation of items parameters of the Mindless Eating subscale.

Item	Factor Loading	a	b1	b2	b3	b4
ME1	0.74	2.782	−2.490	−1.762	−0.912	0.159
ME2	0.63	1.318	−2.816	−1.938	−0.843	0.614
ME3	0.74	2.242	−2.017	−1.232	−0.544	0.354
ME11	0.56	1.416	−2.077	−1.046	−0.149	1.009
ME12	0.53	1.611	−2.939	−2.28	−1.245	−0.039

Note: a: discrimination parameter; b: difficulty threshold parameter.
